# Post-TB lung disease in three African countries

**DOI:** 10.5588/ijtld.22.0261

**Published:** 2022-09-01

**Authors:** A. B. Binegdie, S. Brenac, G. Devereux, H. Meme, A. El Sony, T. H. Gebremariam, R. Osman, B. Miheso, B. Mungai, L. Zurba, M. Lesosky, J. Balmes, P. J. Burney, K. Mortimer, on behalf of the Lung Health in Africa across the Life Course Collaboration

**Affiliations:** 1 Division of Pulmonary and Critical Care Medicine, Department of Internal Medicine, College of Health Sciences, Addis Ababa University, Addis Ababa, Ethiopia; 2 Department of Clinical Sciences, Liverpool School of Tropical Medicine, Liverpool, UK; 3 Centre for Respiratory Diseases Research, Kenya Medical Research Institute (KEMRI), Nairobi, Kenya; 4 Epidemiological Laboratory (Epi-Lab) for Public Health, Research and Development, Khartoum, Sudan; 5 Centre for Health Solutions-Kenya, Nairobi, Kenya; 6 Education for Health Africa, Durban, South Africa; 7 Division of Epidemiology Biostatistics, School of Public Health & Family Medicine, University of Cape Town, Cape Town, South Africa; 8 University of California, San Francisco, CA, USA; 9 National Heart and Lung Institute, Imperial College London, London, UK; 10 Liverpool University Hospitals NHS Foundation Trust, Liverpool, UK; 11 University of Cambridge, Cambridge, UK

Dear Editor,

Chronic respiratory disease (CRD) after pulmonary TB is a recognised contributor to excess morbidity after TB treatment completion, and associations between previous TB disease and abnormal lung structure and function have been consistently demonstrated.^1^–^5^ In recent years, there has been renewed focus on the high burden and impact of post-TB lung disease (PTLD) for patients, their families and communities, particularly in low- and middle-income countries (LMICs).^6^–^9^ There has, however, been less focus on the impact of previous TB on the clinical case load of health services in LMIC settings. We recently characterised the common CRDs encountered in hospital outpatient clinics in three African countries.^10^ We report here on the clinical characteristics of people with previous TB attending hospital-based clinics in three sub-Saharan LMICs: Ethiopia, Kenya and Sudan.

The study methodology has been described in detail elsewhere.^10^ In brief, consecutive adult patients aged ≥18 years with CRD symptoms (>8 weeks) were recruited when attending outpatient departments of general hospitals in three sub-Saharan African countries: Bishofitu Hospital, Addis Ababa, Ethiopia; Mbagathi Hospital, Nairobi, Kenya; and Shabb Hospital, Khartoum, Sudan. In 2020, TB incidence rates in these countries were respectively 132, 259 and 63 per 100,000.^11^ Patients were excluded if there was a clinical suspicion of TB, a positive GeneXpert (Cepheid, Sunnyvale, CA, USA) sputum test result or an acute respiratory infection. As described elsewhere, respiratory symptoms and diagnoses were collected by an interviewer who administered a respiratory questionnaire, and preand post-bronchodilator lung function was measured using spirometry, which was subject to quality control and compared against GLI (Global Lung Function Initiative) 2012 reference values.^10^,^12^ Allergen skin prick testing was performed in accordance with European Standards using grass, cat, dog, cockroach and dust mite allergen solutions (ALK-Abelló Ltd, Reading, UK); a patient was considered atopic if at least one skin prick test was positive.^13^ The diagnosis made by the reviewing clinician was also recorded. All participants provided written informed consent.

A total of 519 patients took part (209 in Kenya, 170 in Ethiopia and 140 in Sudan) and their characteristics are described elsewhere.^10^ The median age was 45 years (IQR 31–57); 53.0% were women, 85.3% had never smoked and the most common clinician diagnosis was asthma (35.8%), followed by chronic bronchitis (24.9%) and chronic obstructive pulmonary disease (COPD) (7.9%). In total, 94 (18.1%) patients reported they had been treated for TB in the past; PTLD was the clinician diagnosis for 22 (4.2%), being most common in Sudan (10%) and least common in Ethiopia (0%). Although there were notable significant differences between countries for most parameters, there were no significant differences in patient reports of TB treatment or clinician diagnosis of asthma. The Table presents comparisons of chronically symptomatic patients attending the clinics with and those without a previous history of TB treatment. Most notably, patients with previously treated TB were less likely to have a clinician diagnosis of asthma (23.4% vs. 38.6%) and less likely to report wheezing in the last 12 months (55.3% vs. 73.9%). Although patients with a previous history of TB were more likely to have a productive cough (69.7% vs. 52.9%), they were less likely to have a clinician diagnosis of chronic bronchitis (17.0% vs. 26.6%, *P* = 0.051) and there was no difference in diagnosed bronchiectasis (7.4% vs. 4.9%). Although patients who reported previous treatment for TB were more likely to have a clinician diagnosis of PTLD (20.1%), there were notable between-country differences: in Sudan, 46.2% of those with previous TB had a clinician diagnosis of PTLD; in Kenya, 18.2% and in Ethiopia no patient with previous TB had a clinician diagnosis of PTLD. In total, 426 (82%) patients provided acceptable and repeatable pre- and post-bronchodilator spirometry.^10^ Patients with previously treated TB had significantly reduced FEV_1_ (forced expiratory volume in 1 s) and FVC (forced vital capacity) values; however, a previous history of TB was not associated with an increase in fixed airflow obstruction (COPD) or bronchodilator reversibility. Overall patterns of lung function in patients with previously treated TB were as follows: normal (26.5%), pure obstruction (16.9%), pure restriction (20.5%) and mixed obstructive/restrictive pattern (26.5%); this did not significantly differ from those of patients with no previous history of TB treatment. In total, 428 (82.4%) patients underwent allergen skin prick testing: Ethiopia (100%), Kenya (*n* = 202, 92%) and Sudan (*n* = 56, 40%) were tested. In total, 70.5% (*n* = 302) of patients had at least one positive skin prick test and were considered atopic; there was no significant difference between patients with and those without a previous history of treated TB (*n* = 57, 73.1% vs. *n* = 245, 70.0%, respectively; *P* = 0.590).

**Table:** Comparison of patients with and without previous history of treated TB

	Previous TB treatment (*n* = 94)^*^ *n* (%)	No previous TB treatment (*n* = 425)^*^ *n* (%)	*P* value
Female sex	40 (42.6)	235 (55.3)	0.025
Age, years, median [IQR]	44 [32–58]	45 [31–57]	0.780
Ever smoked	17 (18.5)	63 (15.0)	0.411
Employed	74 (78.7)	267 (62.8)	0.003
Living in rural area	12 (12.8)	61 (14.4)	0.746
Symptoms			
Wheeze in last 12 months^†^	52 (55.3)	314 (73.9)	<0.001
Productive cough^‡^	62 (69.7)	207 (52.9)	0.004
Clinician diagnosis			
Asthma	22 (23.4)	164 (38.6)	0.005
Chronic bronchitis	16 (17.0)	113 (26.6)	0.051
COPD	9 (9.6)	32 (7.5)	0.526
Bronchiectasis	7 (7.4)	21 (4.9)	0.331
PTLD	20 (21.3)	2 (0.5)	<0.001
Clinical results			
FEV_1_ % predicted, mean, % (95% CI)	65.7 (60.4–71.1)	76.1 (73.4–78.8)	0.001
FVC % predicted, mean, % (95% CI)	73.9 (69.3–78.5)	83.5 (81.1–85.8)	<0.001
FEV_1_/FVC, mean (95% CI)	71.7 (68.4–75.0)	73.8 (72.4–75.3)	0.205
FEV_1_/FVC<0.7	31 (36.0)	112 (31.0)	0.440
FEV_1_/FVC<LLN	34 (39.5)	120 (33.2)	0.312
Reversibility	17 (19.8)	92 (25.5)	0.267
Classification of individuals			
Normal	22 (26.5	120 (35.0)	0.548
Pure obstruction	14 (16.9)	64 (18.7	
Pure restriction	17 (20.5)	56 (16.3)	
Mixed obstruction/restriction	22 (26.5)	77 (22.4)	

* Spirometry results (GLI 2012 reference values): previous TB (*n* = 83), no previous TB (*n* = 343).

† Response to ‘Have you had wheezing or whistling in the chest in the past 12 months?’

‡ Response to ‘Do you usually bring up phlegm from your chest, or do you usually have phlegm in your chest that is difficult to bring up when you don’t have a cold?’

IQR = interquartile range; COPD = chronic obstructive pulmonary disease; PTLD = post-TB lung disease; FEV_1_ = forced expiratory volume in 1 s; FVC = forced vital capacity; CI = confidence interval; LLN = lower limit of normal (GLI 2012); GLI = Global Lung Function Initiative.

The majority of the literature on the impact of TB on CRD is community- or TB patient cohort-based. Although our small study of 519 patients with CRD symptoms has obvious limitations, we believe it is the first to investigate the contribution of previous TB in patients with chronic respiratory symptoms presenting to clinics with notably different patient profiles. It is therefore relevant to the real-life health service experience of PTLD in LMICs. Patients who reported being treated for TB in the past were less likely to report current wheezing symptoms and were less likely to have clinician-diagnosed asthma; however previous TB was not associated with atopic status as determined using allergen skin prick testing, suggesting that any association is independent of atopic status and putative effects of mycobacteria on T-helper cell differentiation.^14^ Although patients previously treated for TB were more likely to report productive cough, clinicians were less likely to diagnose chronic bronchitis (*P* = 0.051), and there was no association with clinician diagnosis of bronchiectasis, a well-recognised manifestation of PTLD, probably reflecting a lack of accessible cross-sectional imaging. The absence of association between previous TB and COPD is almost certainly a consequence of the fact that our study investigated symptomatic patients attending hospital-based clinics (i.e., we compared symptomatic post-TB patients with symptomatic patients with no history of TB), but not community- or TB patient cohort-based.

Overall, previous TB contributed to a sizeable minority (about a fifth) of patients attending the clinics. However, there were notable differences in the clinician diagnosis of PTLD, ranging from 0% in Ethiopia to 10% in Sudan, and the proportion of patients with previous TB with clinician-diagnosed PTLD, which ranged from 0% in Ethiopia to 46% in Sudan. Although this variation may reflect differences in local research interests, or diagnostic practices, they certainly highlight the need for widespread implementation of generally accepted diagnostic criteria for PTLD.^15^
